# CD-Loop: a chromatin loop detection method based on the diffusion model

**DOI:** 10.3389/fgene.2024.1393406

**Published:** 2024-05-06

**Authors:** Jiquan Shen, Yang Wang, Junwei Luo

**Affiliations:** School of Software, Henan Polytechnic University, Jiaozuo, China

**Keywords:** chromatin loop, diffusion model, Hi-C contact map, three-dimensional structure, deep learning

## Abstract

**Motivation:**

In recent years, there have been significant advances in various chromatin conformation capture techniques, and annotating the topological structure from Hi-C contact maps has become crucial for studying the three-dimensional structure of chromosomes. However, the structure and function of chromatin loops are highly dynamic and diverse, influenced by multiple factors. Therefore, obtaining the three-dimensional structure of the genome remains a challenging task. Among many chromatin loop prediction methods, it is difficult to fully extract features from the contact map and make accurate predictions at low sequencing depths.

**Results:**

In this study, we put forward a deep learning framework based on the diffusion model called CD-Loop for predicting accurate chromatin loops. First, by pre-training the input data, we obtain prior probabilities for predicting the classification of the Hi-C contact map. Then, by combining the denoising process based on the diffusion model and the prior probability obtained by pre-training, candidate loops were predicted from the input Hi-C contact map. Finally, CD-Loop uses a density-based clustering algorithm to cluster the candidate chromatin loops and predict the final chromatin loops. We compared CD-Loop with the currently popular methods, such as Peakachu, Chromosight, and Mustache, and found that in different cell types, species, and sequencing depths, CD-Loop outperforms other methods in loop annotation. We conclude that CD-Loop can accurately predict chromatin loops and reveal cell-type specificity. The code is available at https://github.com/wangyang199897/CD-Loop.

## 1 Introduction

The genome of eukaryotic organisms exists in the form of nuclear chromatin, and the function of chromatin is closely related to its three-dimensional structure. For example, biological functions such as genome replication, transcription, regulation, DNA mutation, the spread of long non-coding RNA, and embryonic development all are completed in the three-dimensional space of the cell nucleus (Bonev and Cavali, 2016). In recent years, with the development of high-throughput chromosome conformation capture (Hi-C) (Lieberman-Aiden et al., 2009) and chromatin interaction analysis by paired-end tag sequencing (ChIA-PET) technologies (Fullwood et al., 2009), researchers have discovered that chromosomes can be categorized into chromatin compartments (A/B compartments), topologically associated domains (TADs), and chromatin loops. Chromatin loops, such as enhancer-promoter loops, explain the regulatory mechanism of enhancers on target genes. Despite the enhancer being far away from the target gene in linear distance, the enhancers and target gene promoters are located on the same chromatin loop in close spatial proximity, regulating the target gene by binding to the promoter (Dekker et al., 2013; Dixon et al., 2015; Gorkin et al., 2014; Rao et al., 2014; Dixon et al., 2012).

The chromatin loop is an advanced structural form of chromatin in eukaryotic organisms. In previous studies, chromatin loops could not be observed, but with the emergence of three-dimensional structures, it is now possible to clearly observe various organizations of genes. Experiments have shown that the chromatin loops are linked to proteins such as CTCF and cohesin. Two genes may be linearly distant from each other, but their spatial arrangement is not linear, and their spatial distance may be very close. Therefore, the two genes may interact with each other. They may be close in spatial proximity, potentially allowing for interactions between the two genes. We call the loop-like structure formed by two genes that are close together and the chromatin segment between them a chromatin loop. During the formation of cancer, the structure of chromatin loops may also undergo changes, leading to alterations in cancer-related genes (Wang et al., 2022). In genome-wide association studies (GWAS), it has been discovered that certain immune-related genetic variations are concentrated in chromatin loops specific to blood cells rather than embryonic cells, indicating that these chromatin loops can help us further understand certain disease variations (Buenrostro et al., 2013; Tang et al., 2015; Szabo et al., 2019; Grubert et al., 2020; Kloetgen et al., 2020).

Although some important progress has been made in the study of chromatin loops, the structure and function of chromatin loops are highly dynamic and diverse and are influenced by various factors. Currently, our comprehension of the structure and role of chromatin loops in the three-dimensional space of the cell nucleus remains limited, rendering it challenging to anticipate the consequences of alterations in the chromatin loop structure on gene mutations. Therefore, it remains a challenging problem to acquire the correlation between the three-dimensional architecture and functionality of the genome and use experimental techniques to detect chromatin loops in cell types or species with unknown 3D structures. At low coverage, due to the limited amount of data and the presence of random noise and biases, the detection of loops will be more challenging. Therefore, more accurate and efficient computational models and methods are needed to address these issues. This will help us better understand the organization, function, and gene regulation mechanisms of chromatin loops.

The methods for predicting chromatin loops are diverse, mainly encompassing the following aspects: (1) prediction of chromatin loops based on statistical methods. For high-throughput chromosome conformation capture (Hi-C), it focuses on the entire cell nucleus, studies the spatial relationships of the entire chromatin across the whole genome, and achieves the capture of interactions between chromatin segments across the entire genome. The corresponding tools are as follows: HiCCUPS (Rao et al., 2014; Durand et al., 2016) integrates nearby background information into its framework and employs a Poisson test in conjunction with an adapted Benjamini–Hochberg procedure to assess the significance of chromatin interactions. The HiCExplorer method (Wolff et al., 2022) uses ongoing negative binomial distribution and the Wilcoxon rank-sum test to ascertain the enrichment of Hi-C interactions by considering the neighborhood of candidate elements and distinguishing significant peaks from background noise. For ChIA-PET technology, using PET sequencing technology to study DNA fragments with nearby connections after immunoprecipitation allows researchers to obtain chromatin interactions; this fundamentally investigates the interactions between DNA fragments. The difference between Hi-C and ChIA-PET lies in the fact that data generated by Hi-C reflect chromatin interactions, including all proteins, while the ChIA-PET technology enrichment of specific protein factors results in data that represent chromatin interactions of a particular protein. Using ChIA-PET technology to develop tools includes the ChIA-PET tool (Li et al., 2010), which employs the hypergeometric distribution to filter noise. Mango software (Phanstiel et al., 2015) establishes a null model by merging the genomic distances and read depths for each anchor point. For the capture Hi-C technique (Mifsud et al., 2015), an additional capture step is introduced on top of the traditional Hi-C library preparation process to capture target fragments for subsequent sequencing. CHiCAGO (Cairns et al., 2016) employs an innovative background correction technique and a two-component convolution background model while addressing multiple testing through a *p*-value weighting approach. The ChiCMaxima method (Ben Zouari et al., 2019) applies loess smoothing to the captured Hi-C reads and transforms the detection of chromatin loops into the search for peaks from the loess-smoothed profiles. HiChIP (Mumbach et al., 2016) is a protein-centric approach for studying chromatin conformation, which synergistically combines Hi-C technology and ChIA-PET technology to extract more detailed three-dimensional chromatin structure information using a reduced dataset. Related tools include HiChIP-Peaks (Shi et al., 2020), which models the background signal as a negative binomial to simulate excessive dispersion and identify enriched signal regions. It also corrects HiChIP specific biases caused by the uneven distribution of restriction enzyme sites. (2) Prediction of chromatin loops based on traditional methods. Lollipop (Kai et al., 2018) is a machine learning framework based on the random forest classifier, which uses genomic and epigenomic features to predict CTCF-mediated interactions. CTCF-MP (Zhang et al., 2018), based on word2vec and boosted trees, accurately predicts loops formed by convergent CTCF motifs using sequence features, CTCF ChIP-seq and DNase-seq. C-Loops (Cao et al., 2020) relies on the clustering algorithm cDBSCAN, which directly examines paired-end tags (PET) to detect potential loops and employs permuted local backgrounds to estimate their significance. However, one of the recent trends in research is to apply computer vision and machine learning techniques to the annotation of topological structures. For example, the SIP method (Rowley et al., 2020) applies Gaussian smoothing, contrast adjustment, morphological white top-hat transformation, and a maximum–minimum filter to an image. After these steps, the corrected image of the interaction is provided, which is used in conjunction with the regional maxima detection algorithm to detect loops. Peakachu (Salameh et al., 2020) uses a classification framework to forecast chromatin loops based on the Hi-C contact map, capable of identifying a unique set of short-range interactions. Chromosight (Matthey-Doret et al., 2020) is a computer vision-based algorithm that takes whole-genome contact matrices as the input and uses a balancing normalization procedure to mitigate experimental biases. The Mustache method (Roayaei Ardakany et al., 2020) represents the interaction matrix using scale-space theory, and we consider the identification of chromatin loops as a problem of detecting spot-like objects. Both of these pattern-based general methods work well with a sufficient number of contact pairs but perform poorly at low sequencing depths. Due to the swift advancement and widespread utilization of deep learning technology, significant progress has been made in bioinformatics. It is not surprising that some work has been achieved in the field of genomics. For example, DeepLUCIA (Yang et al., 2022), a deep learning-based chromatin interaction model, utilizes epigenomic information to forecast chromatin loops in various tissues. The predicted chromatin loops can help enhance our understanding of the genomic structure of human tissues. DeepMILO (Trieu et al., 2020) uses a deep learning framework to anticipate the impacts of mutations on CTCF-mediated insulator loops. DeepLoop (Zhang et al., 2022) discovers noteworthy interactions from Hi-C contact maps using neural networks to denoise and enhance loop signals. RefHiC (Zhang and Blanchette, 2022) is a deep learning method that uses high-quality Hi-C datasets with different cell types to study the topological structure annotation of samples. GILoop (Wang et al., 2022) is a twin-branch neural network that utilizes the image view and graph view to identify interactions in the entire genome. Be-1DCNN (Wu et al., 2023) utilizes a bagging ensemble learning strategy and one-dimensional convolutional neural network (1DCNN) to improve the accuracy and reliability of predictions by integrating multiple 1DCNN models.

Although some progress has been made with the above methods, it remains a significant challenge to fully extract features from Hi-C contact maps and identify chromatin loops in different sequencing depths and cell lines. Recently, the denoising diffusion probabilistic model (DDPM) (Ho et al., 2020) has had good performance in image generation and synthesis tasks by progressively enhancing the quality of the provided image. Furthermore, the diffusion model is able to simulate the propagation and influence of features in an image, which helps better capture local and global features, thus improving classification accuracy (Han et al., 2022). Here, we propose a method, named CD-Loop, based on the diffusion model, which combines CTCF ChIA-PET and H3K27ac HiChIP (Mumbach, 2017) data derived from biologically diverse experiments to label samples. This approach aims to cover a wider range of chromatin loops. Using a conditional generative model based on noise addition and noise reduction, along with a pre-trained conditional mean estimator, we convert the task of identifying chromatin loops into a binary classification task. The results indicate that training the data only on the original sequencing depth is effective for different cell types, sequencing depths, and species with high precision and recall. In comparison to existing methods, our approach successfully identifies a set of distinct chromatin loops.

## 2 Materials and methods

**FIGURE 1:**
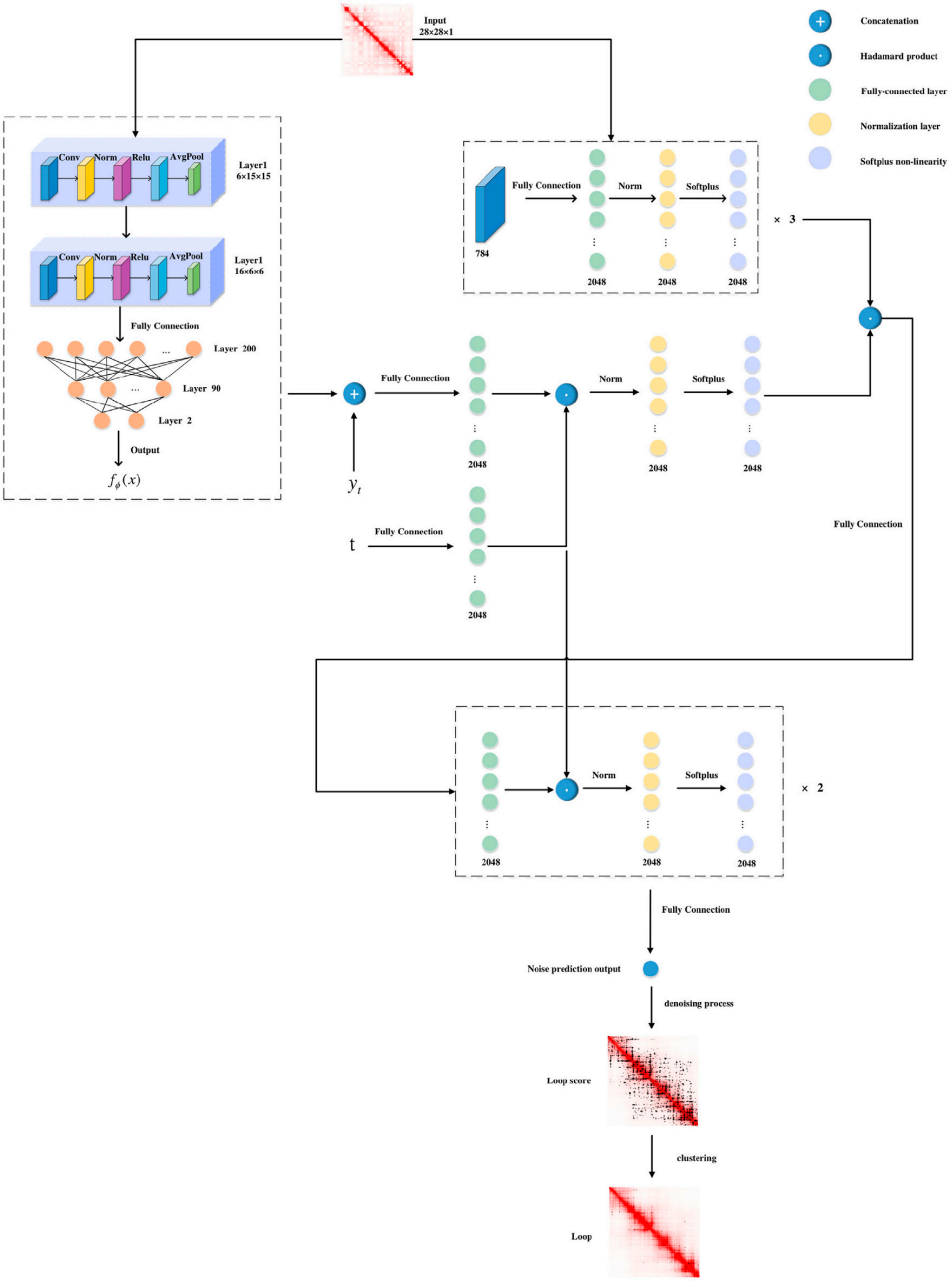
CD-Loop architecture. Overview of the CD-Loop neural network for loop.

CD-Loop takes a Hi-C contact map as the input and predicts highly reliable chromatin loops. The model can be roughly divided into two parts, as shown in Figure 1. (1) First, CD-Loop pre-trains the input data using the LeNet5 model to obtain the prior probability of predicted classification for the input Hi-C contact map. (2) Then, by combining the denoising process based on the diffusion model and the prior probability obtained by pre-training to predict the candidate chromatin loop, the output includes the probability score, CI confidence, and two-tailed t-test evaluation metrics for each candidate chromatin loop. (3) Finally, the low-scoring candidate chromatin loop is filtered out, and then clustering is performed based on the density algorithm to select representative chromatin loops.

### 2.1 Pretraining phase

The CD-Loop network first applies a pre-trained conditional mean estimator, utilizing the LeNet5 network, with an input of dimension of 2w × 2w, where w is the window size (w = 14). This module consists of two convolutional blocks, three fully connected layers, and two ReLU layers. Each block includes a convolution operation, batch normalization, a ReLU activation function, and an average pooling operation. The input and output of this process are class labels, which we refer to as prior probabilities.

### 2.2 Diffusion model

The second part of the model applies the forward and backward processes of the diffusion model, assuming that the endpoint of our forward process is

$$p\left(y_{T}|x\right)=N\left(f_{\emptyset }\left(x\right),I\right),$$

where $f_{\emptyset }\left(x\right)$ is the prior probability with respect to x and $y_{0}$ . The conditional distribution of the forward process can be defined as follows for all timesteps including t=1 :

$$q\left(y_{t}|y_{t-1},f_{\emptyset }\left(x\right)\right)=N\left(y_{t};\sqrt{1-\beta_{t}}y_{t-1}+\left(1-\sqrt{1-\beta_{t}}\right)f_{\emptyset }\left(x\right),\beta_{t}I\right),$$

which enables a closed sampling distribution with arbitrary timesteps t:

$$q\left(y_{t}|y_{0},f_{\emptyset }\left(x\right)\right)=N\left(y_{t};\sqrt{{\bar{\alpha }}_{t}}y_{0}+\left(1-\sqrt{{\bar{\alpha }}_{t}}\right)f_{\emptyset }\left(x\right),\left(1-{\bar{\alpha }}_{t}\right)I\right),$$

where $\alpha_{t}:=1-\beta_{t}$ and ${\bar{\alpha }}_{t}:=\Pi_{t}\alpha_{t}$ . The backward process can be defined as follows:

$$p\left(y_{t-1}|y_{t},y_{0},x\right)=p\left(y_{t-1}|y_{t},y_{0},f_{\emptyset }\left(x\right)\right)=N\left(y_{t-1};\tilde{\mu }\left(y_{t},y_{0},f_{\emptyset }\left(x\right)\right),{\tilde{\beta }}_{t}I\right),$$

where

$$\tilde{\mu }:=\underset{\gamma_{0}}{\underbrace{\frac{\beta_{t}\sqrt{{\bar{\alpha }}_{t-1}}}{1-{\bar{\alpha }}_{t}}}}y_{0}+\underset{\gamma_{1}}{\underbrace{\frac{\left(1-{\bar{\alpha }}_{t-1}\right)\sqrt{\alpha_{t}}}{1-{\bar{\alpha }}_{t}}}}y_{t}+\underset{\gamma_{2}}{\underbrace{\left(1+\frac{\left(\sqrt{{\bar{\alpha }}_{t}}-1\right)\left(\sqrt{\alpha_{t}}+\sqrt{{\bar{\alpha }}_{t-1}}\right)}{1-{\bar{\alpha }}_{t}}\right)}}f_{\emptyset }\left(x\right),$$

$${\tilde{\beta }}_{t}:=\frac{1-{\bar{\alpha }}_{t-1}}{1-{\bar{\alpha }}_{t}}\beta_{t}.$$

After organizing the process, the optimization objective is the maximization of the likelihood function:

$$\log p_{\theta }\left(y_{0}|x\right)=\log\int p_{\theta }\left(y_{0:T}|x\right)dy_{1;T}\geq E_{q\left(y_{1:T}|y_{0},x\right)}\left[\log\frac{p_{\theta }\left(y_{0:T}|x\right)}{q\left(y_{1:T}|y_{0},x\right)}\right].$$

We chose the diffusion model as the second part of our model because through the iterative process of the diffusion model, the noise in the Hi-C interaction matrix can be corrected, and the quality is improved. In addition, the diffusion model can simulate the spread and influence of features in the image, helping better capture the local and global features in the Hi-C interaction matrix, thereby improving the accuracy of the classification.

This part of the model framework applies an encoder to a flattened input image to obtain a 2048-dimensional representation. The encoder consists of three fully connected layers, with an output size of 2048. Meanwhile, we concatenate $y_{t}$ and the output $f_{\emptyset }\left(x\right)$ from the first part, applying a fully-connected layer to generate an output vector of 2048 dimensions. To incorporate the timestep information, we apply a Hadamard product between the vector and timestep embedding, generating a response embedding specific to the timestep. Next, we integrate this response embedding with the image embedding through another Hadamard product. The resulting vector is then passed through two additional fully-connected layers. Each layer has 2048 dimensions. Before each layer, a Hadamard product is performed with timestep embedding. At last, a fully-connected layer is employed to predict. It is worth noting that, in addition to the output layer, there is also a batch normalization layer and Softplus non-linearity after each fully connected layer. The architecture is shown in Table 1.

**TABLE 1:** Diffusion model network architecture.

$\text{input}:x,y_{t},f\varphi \left(x\right),t$
$l_{1,x}=\sigma \left(\text{BN}\left(g_{1,x}\left(x\right)\right)\right)$
$l_{2,x}=\sigma \left(\text{BN}\left(g_{2,x}\left(x\right)\right)\right)$
$l_{3,x}=\text{BN}\left(g_{1,x}\left(x\right)\right)$
$l_{1,y}=\sigma \left(\text{BN}\left(g_{1,y}\left(y_{t}⊕f_{\emptyset }\left(x\right)\right)⊙g_{1,b}\left(t\right)\right)\right)$
$l_{1}=l_{3,x}⊙l_{1,y}$
$l_{2}=\sigma \left(\text{BN}\left(g_{2,a}\left(l_{1}\right)⊙g_{2,b}\left(t\right)\right)\right)$
$l_{3}=\sigma \left(\text{BN}\left(g_{3,a}\left(l_{2}\right)⊙g_{3,b}\left(t\right)\right)\right)$
$\text{output}:g_{4}\left(l_{3}\right)$

⊕: concatenation; ⊙: Hadamard product; σ: Softplus non-linearity; g: a fully-connected layer; and l: a hidden layer output.

The model framework outputs a noise prediction and then utilizes a denoising process, combined with pre-trained prior probabilities, to obtain the posterior mean and posterior variance. Based on the obtained posterior mean and posterior variance, the predicted label at time T-1 is calculated from time T, and this process is repeated until time 1.

### 2.3 Detect loops by density-based clustering

For the window centered around each bin pair (i, j) after model prediction, CD-Loop generates a probability score s (i, j) for each bin pair. A higher score value indicates a higher likelihood of the bin pair being a loop. Therefore, we retain bin pairs that are predicted as loops and have a score greater than 0.5, and these bin pairs (i, j) are referred to as candidate loops. If there are fewer than 15 candidate loops within a 5-bin by 5-bin square centered around (i, j), it is referred to as an isolated prediction. These isolated predictions are likely to be false positives and are therefore excluded. Then, we use a density-based clustering algorithm to cluster the remaining candidate loops. First, we use the nearest neighbors (Abeywickrama et al., 2016) method to compute the local density of each candidate (i , j). To achieve a fast nearest neighbor search, we use the K-D tree data structure, and the distance metric used is Chebyshev distance. We then calculated and recorded the indices and distances of the nearest neighbors for each candidate (i, j). By iterating over the nearest neighbors of each candidate (i, j), find the nearest neighbor with a higher density than itself. If the nearest neighbor with a higher density than the current point is found, we record its index and distance as the delta value. If no such point is found, meaning that the candidate (i, j) has the highest local density within the current range, we set the delta value to a distance greater than that of the neighboring nodes. We repeat this process, increasing the query radius until the nearest neighbors of all candidate (i, j) pairs are found. Finally, we discard candidate loops with delta values less than 5, as they may represent redundant predictions. The remaining candidate loops after filtering constitute our final predicted loops. The same parameters are used in different datasets and different coverages, and these parameters perform well in the final prediction.

### 2.4 Composition of training samples

Selection of positive samples: CD-Loop selects the combination of CTCF ChIA-PET data and H3K27ac HiChIP data and then removes all interaction pairs outside the range of 30 kb to 3 Mb as positive sample data. Because in loop annotation, CTCF ChIA-PET data contain long-range interactions, while H3K27ac HiChIP data contain shorter-range interactions, combining the two can cover a wider range of loop types.

Selection of negative samples: Due to a large unbalance in the number of positive and negative samples, we selected different types of negative samples three times based on the genomic distance characteristics of the positive samples, ensuring that the genomic distance of each negative sample falls within the range of 30 kb to 3 Mb. This can reduce the number of negative samples and consider all the characteristics of negative samples as much as possible. (1) For each positive interaction pair, two negative interaction pairs with the same genomic distance are randomly selected from the entire genome. (2) For all possible genomic distances of positive samples, randomly select a genomic distance each time, and then a negative interaction pair with the same genomic distance is randomly selected from the entire genome until the number of generated negative samples equals that of positive samples. (3) For the largest genomic distance among positive samples, a value greater than that distance is randomly chosen as the genomic distance for negative samples, and a negative interaction pair is randomly selected from the entire genome until the number of generated negative samples equals that of positive samples.

We selected positive and negative samples within the gene distance range of 30 kb to 3 Mb for the following two reasons: first, interactions that are far apart in the genome are more likely to have sequencing errors, resulting in chromatin loops that are detected in distant genomes having high error rates. Interactions that are relatively close together are generally caused by physical interactions rather than true chromatin loops. Second, in other chromatin loop prediction methods, most of the predicted chromatin loops are in the range of 30 kb to 3 Mb. So, considering these two factors, we chose the range of 30 kb to 3 Mb to filter other samples.

Data preprocessing: The input Hi-C contact map is divided into bins at a resolution of 5 kb. Due to the existing sampling bias and technical noise, Knight–Ruiz (KR) normalization (Knight and Ruiz, 2013) is used for correction. If a positive sample is represented by two or more pixels in the contact map, each pixel represents a positive interaction pair. After obtaining the positive and negative interaction pairs, in the Hi-C contact map, with each interaction pair as the center, 13 bins are selected upward and to the right, and 14 bins are selected to the right and downward, forming a 28*28 matrix. The matrices from the negative sample matrix that consist entirely of zero elements are removed. The reasons why we delete matrices with all 0 elements in the negative sample matrix are as follows: first, consider that in the Hi-C interaction matrix, all elements are 0, which means that there is no interaction between one region of the chromosome and another region. Such a matrix does not contain any meaningful information. Second, the model cannot extract effective features from these matrices with all 0 elements. There are too many such matrices in the training set, which will only increase the training time.

### 2.5 Model training and prediction

During the model training and testing process, Hi-C contact maps from the GM12878 dataset are adopted. For validation, we used chr11 and chr12, while chr15, chr16, and chr17 were used for testing and prediction. The remaining chromosomes were used for training. During the model training process, data augmentation was performed by flipping the 28*28 matrices generated from the positive samples horizontally and vertically. In the model prediction process, since it involves taking every bin pair of an entire chromosome as the input, the amount of data is very large. Therefore, we performed the following three preprocessing steps on the chromosome to be predicted. (1) The genomic distance threshold between bin pairs: since the distribution of chromatin loops in the genome ranges from approximately 30 kb to 3 Mb, we remove the predicted bin pairs with a genomic distance greater than 3 Mb or less than 30 kb. (2) Interaction frequency threshold of bin pairs: by observing the interaction frequency of each bin pair in the positive samples, we found that 99% of the positive samples have an interaction frequency greater than 1. Therefore, we remove the bins with an interaction frequency less than 1. (3) Threshold of the number of zero elements in matrices: after counting the number of zero elements in the 28*28 matrices of positive samples, it was found that 90% of the positive sample matrices have less than 200 zero elements. When making predictions for downsampling data, the same processing is applied.

## 3 Results

CD-Loop is trained on the original sequencing depth of GM12878, which is not specific to this sequencing depth or cell type. Next, we will demonstrate the superiority of this model by demonstrating the ability of the same model weights to annotate loops in the same cell type at different sequencing depths. Meanwhile, we will highlight that the model can accurately annotate loops in numerous other cell types without requiring retraining. Furthermore, the same trained model can also be used to annotate loops in mouse Hi-C contact maps. This demonstrates that the CD-Loop model has good generalization and adaptability and is applicable to annotation tasks of different sequencing depths and cell types. In our experiment, chromosomes 11 and 12 are used for validation, chromosomes 15–17 are used for testing, and the remaining chromosomes are used for training. The reported human gene results only apply to the three test chromosomes, while the results for mice apply to all chromosomes.

This method is trained and predicted on RTX4090 GPU and requires at least 15 GB of space to load samples during prediction. The runtime of chromatin loop identification depends on the sequencing depth of Hi-C data. For example, prediction can be completed within 325 min on Hi-C data containing 500 M valid read pairs. The data used in the experiments of this paper are shown in Table 2.

**TABLE 2:** Different sources of datasets.

Deposited data	Source	Identifier	Link
GM12878 Hi-C	GEO	GSE63525	https://www.ncbi.nlm.nih.gov/geo/query/acc.cgi?acc=GSE63525
K562 Hi-C	GEO	GSE63525	https://www.ncbi.nlm.nih.gov/geo/query/acc.cgi?acc=GSE63525
IMR90 Hi-C	GEO	GSE63525	https://www.ncbi.nlm.nih.gov/geo/query/acc.cgi?acc=GSE63525
mESC Hi-C	4D Nucleome	4DNFIU8AF5ZY	https://data.4dnucleome.org/experiment-set-replicates/4DNESUCLJAZ8
GM12878 CTCF ChIP-Seq	ENCODE	ENCFF963PJY	https://www.encodeproject.org/files/ENCFF963PJY
K562 CTCF ChIP-Seq	ENCODE	ENCFF085HTY	https://www.encodeproject.org/files/ENCFF085HTY
IMR-90 CTCF ChIP-Seq	ENCODE	ENCFF453XKM	https://www.encodeproject.org/files/ENCFF453XKM
mESC CTCF ChIP-Seq	ENCODE	ENCFF508CKL	https://www.encodeproject.org/files/ENCFF508CKL
K562 CTCF ChIA-PET	ENCODE	ENCFF001THV	https://www.encodeproject.org/files/ENCFF001THV
K562 RAD21 ChIA-PET	ENCODE	ENCFF002ENT	https://www.encodeproject.org/files/ENCFF002ENT
mESC CTCF ChIA-PET	ENCODE	ENCFF550QMW	https://www.encodeproject.org/files/ENCFF550QMW
GM12878 CTCF ChIA-PET	Reference (Tang et al., 2015)	Tang, Z. et al. (2015)	https://doi.org/10.1016/j.cell.2015.11.024
GM12878 RAD21 ChIA-PET	Reference (Heidari et al., 2014)	Heidari et al. (2014)	https://doi.org/10.1101/gr.176586.114
GM12878 H3k27ac HiChIP	Reference (Mumbach et al., 2017)	Mumbach et al. (2017)	https://doi.org/10.1038/ng.3963
GM12878 SMC1 HiCHIP	Reference (Mumbach et al., 2016)	Mumbach et al. (2016)	https://doi.org/10.1038/nmeth.3999

### 3.1 GM12878 experimental results

We first evaluated the prediction accuracy of chromatin loops by CD-Loop on the original sequencing depth Hi-C dataset (2600 M valid read pairs) from the human GM12878 cell line. Simultaneously, we compared it with several popular methods, including Chromosight, Peakachu, and Mustache. To ensure a fair comparison, we evaluated the chromatin loops at a 5 kb resolution and annotated them from the same dataset using default parameters. Additionally, we applied a consistent 5% FDR cutoff for all tools.

#### 3.1.1 Quantitative analysis

**FIGURE 2:**
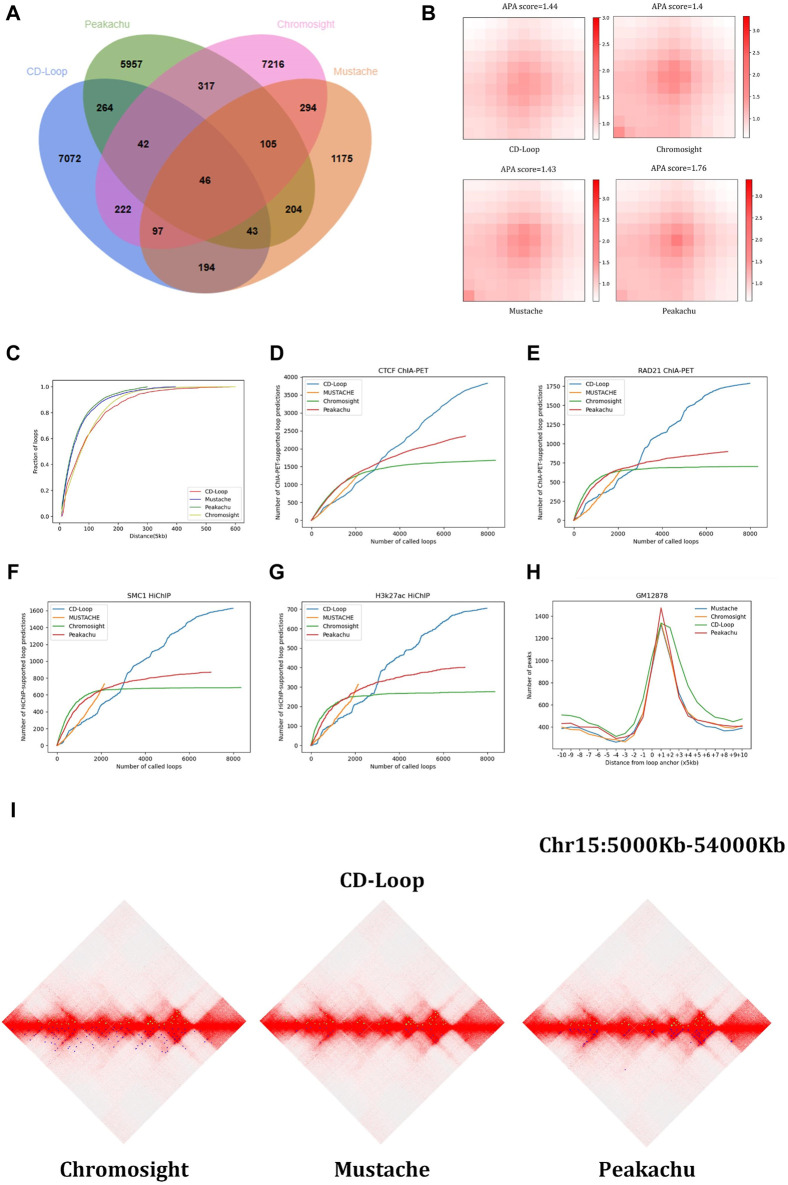
Comparison based on the GM12878 dataset: **(A)** Venn diagram, **(B)** aggregated peak analysis, and **(C)** cumulative distance distribution. The chromatin loop genome distance distribution predicted by CD-Loop is highly similar to that predicted by Chromosight. **(D–G)** Supporting loops validated by CTCF ChIA-PET **(D)**, RAD21 ChIA-PET **(E)**, SMC1 HiChIP **(F)**, and H3k27ac HiChIP **(G)** enrichment experiments for loops predicted by CD-Loop and other tools. The loop predictions by CD-Loop align better with these experimental data compared to other tools on the testing chromosomes. **(H)** Function depicting the distance from predicted loop anchors to CTCF-binding sites identified by ChIP-seq signals. **(I)** Visualization example of loop identification. The upper half of the three diamond plots display green dots, which represent CD-Loop. On the other hand, the lower half of the plots consists of blue dots, which represent Chromosight, Mustache, and Peakachu, respectively.

Loops predicted by different tools vary significantly. First, among the four methods, we controlled the genomic distance of all predicted chromatin loops between 30 kb and 3 Mb. To compare the performance with all methods, we ensured that the anchoring points of two chromatin loops fully matched and overlapped. As shown in Figure 2A, we found that CD-Loop identified almost as many chromatin loops as Chromosight and Peakachu. For the results, 89% of CD-Loop, 87% of Chromosight, 85% of Peakachu, and 54% of Mustache were unique.

#### 3.1.2 Aggregation peak analysis

The aggregation peak analysis of the four methods in the GM12878 cell line is shown in Figure 2B. The APA score quantifies the loop pattern of detected peaks by comparing the number of reads in the center point bin to the average number of reads in the lower left corner of the matrix. We only consider the top 2,000 loops with high scores. Compared with Peakachu, the loops detected by Chromosight, CD-Loop, and Mustache have more dispersed loop centers, and the three methods have a similar APA score. Since we split the chromatin loop spanning multiple pixel points into multiple chromatin loops, each pixel is regarded as a single chromatin loop, and the most representative pixel among them is not selected as a positive sample. So, the boundary range of chromatin loops is expanded, and the detected chromatin loops have dispersed loop centers. Next, we compared the genomic distances of loop anchors predicted by the four methods. As shown in Figure 2C, the distance distributions between chromatin loop anchors predicted by CD-Loop and Chromosight are similar and have larger genetic distances, while Peakachu and Mustache predict more short-range interactions.

#### 3.1.3 Enrichment experimental analysis

Then, we compared the chromatin loops predicted by different methods on different datasets. Different enrichment experimental data include CTCF ChIA-PET, RAD21 ChIA-PET, SMC1 HiChIP, and H3k27ac HiChIP. We make predictions for the three chromosomes chr15, chr16, and chr17 of the test set and compare them with three other methods to evaluate these loops (allowing an error of 5 kb). As shown in Figures 2D–G, among the four methods, Mustache predicts the smallest number of loops and the least number of overlaps with enrichment experiments. The remaining three methods predict almost the same number of loops, but CD-Loop has the largest number of correct predictions across different enrichment experiments and has the highest recall rates. CD-Loop predicted a total of 7,980 loops, with 3,821 correctly predicted loops in the CTCF dataset, yielding an accuracy of 48%. Peakachu predicted 6,978 loops, with 2,351 correctly predicted loops in the CTCF dataset, resulting in an accuracy of 33%. Chromosight predicted 8,993 loops, with 1,676 correctly predicted loops in the CTCF dataset, giving an accuracy of 20%. Mustache predicted 2,158 loops, with 1,293 correctly predicted loops in the CTCF dataset, achieving the highest accuracy of 59%. CD-Loop had the highest number of successfully predicted loops, ranking second in accuracy, while Mustache, with the highest accuracy, had the fewest successfully predicted loops, only one-third of CD-Loop’s count. The specific data for RAD21, SMC1, and H3K27ac can be found in Figures 2E–G, where CD-Loop demonstrates good performance in both accuracy and recall.

#### 3.1.4 CTCF-binding site analysis

We next performed this by visualizing the CTCF ChIP-Seq and H3k27ac HiChIP-binding signals on the flanking regions around the loop anchors. As shown in Figure 2H, the predicted loop anchors detected by CD-Loop showed a clear enrichment effect in CTCF, and the H3k27ac-binding motif proves that CD-Loop can not only identify loops related to CTCF but also loops related to H3k27ac.

#### 3.1.5 Hi-C heat map analysis

The genome-wide analysis described above demonstrates the good ability of CD-Loop to identify loops in Hi-C contact maps. We used the Juicebox tool (Durand et al., 2016) to visualize chromatin loops for the purpose of visual representation, demonstrating that CD-Loop can detect more chromatin loops and unique chromatin loops undetectable by other methods. As shown in Figure 2I, the upper part is the visual representation of chromatin loops detected by CD-Loop in the Hi-C interaction matrix, and the lower part is the detection of the remaining three methods (Peakachu, Chromosight, and Mustache) for the visual representation of chromatin loops in the Hi-C interaction matrix. The green dots represent the position of the chromatin loop detected by CD-Loop in the Hi-C interaction matrix, and the blue dots represent the position of the chromatin loop detected by Peakachu, Chromosight, and Mustache in the Hi-C interaction matrix. We can find that the results of CD-Loop mostly overlap with the results of other methods, but some are unique.

Taken together, these results show that CD-Loop has better overall prediction accuracy for GM12878 data (2600 M read pairs) than other methods.

### 3.2 Experimental results on other cells and species

Our method was trained on the original test depth data of the human GM12878 cell line, but our findings reveal that the trained model demonstrates better performance across various cell types. To further verify the performance of CD-Loop, we compared CD-Loop and other methods using K562 and IMR90 cell lines from humans (only chromosomes 15–17 test) and mouse embryonic stem cells (mESCs) (all chromosomes).

#### 3.2.1 Number analysis

**FIGURE 3:**
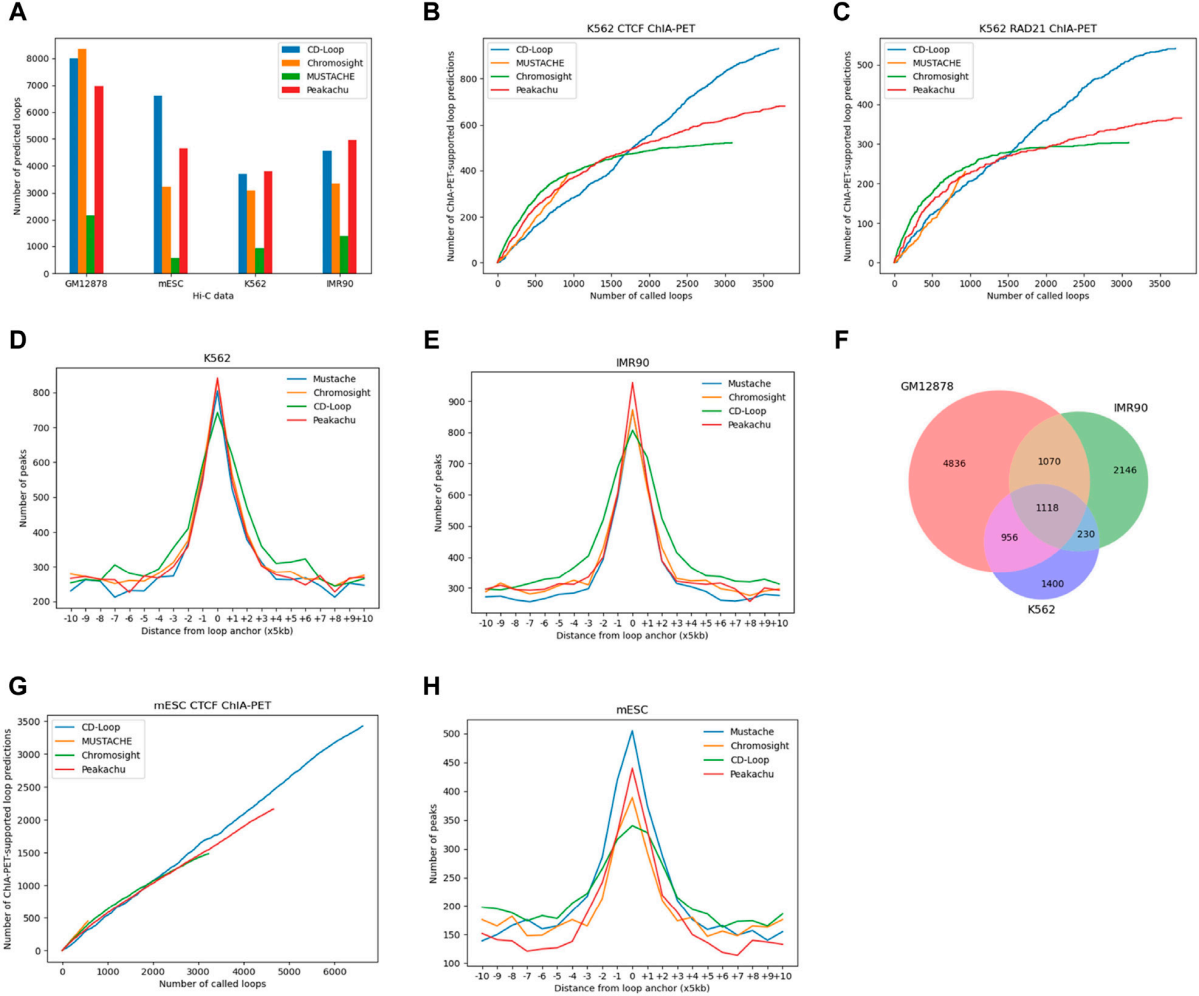
Comparison based on Hi-C data from human K562, IMR90, and mouse ESC. **(A)** The number of loops present. **(B–C)** Overlap between the chromatin loops predicted by CD-Loop and other tools on the K562 Hi-C contact map with CTCF ChIA-PET **(B)** and RAD21 ChIA-PET **(C)** enrichment experiments on testing chromosomes chr15-17. **(D)** Function depicting the distance from predicted loop anchors to CTCF-binding sites in K562 cells identified by ChIP-seq signals. **(E)** Function depicting the distance from predicted loop anchors to CTCF-binding sites in IMR90 cells identified by ChIP-seq signals. **(F)** Venn diagram showing the chromatin loops identified by CD-Loop across three cell lines: GM12878, IMR90, and K562. **(G)** Overlap between the chromatin loops predicted by CD-Loop and other tools on the mESC with CTCF ChIA-PET. **(H)** Function depicting the distance from predicted loop anchors to CTCF-binding sites in mESC cells identified by ChIP-seq signals.

As shown in Figure 3A, in different cell lines of both humans and mice, CD-Loop and Peakachu predicted the most loops, indicating that the CD-Loop method is more reliable for predicting chromatin loops, regardless of sequencing coverage. When applied to the complete set of autosomes with 124 M read pairs from mESC data, the CD-Loop model trained with the GM12878 original sequencing depth was used to predict mouse cell lines. CD-Loop identified a higher number of loops compared to other tools in low-coverage data.

#### 3.2.2 K562 and IMR90 cell line enrichment experiments and CTCF-binding site analysis

Different enrichment experiments were used to reveal loops and evaluate the accuracy of these tools. Like GM12878 before, we controlled the FDR of other methods to 5%. For K562 data, as shown in Figure 3B, CD-Loop predicted a total of 3,704 loops in the K562 cell line, with 1,169 correctly predicted loops in the CTCF dataset, yielding an accuracy of 32%. Peakachu predicted 3,785 loops, with 830 correctly predicted loops in the CTCF dataset, resulting in an accuracy of 22%. Chromosight predicted 3,089 loops, with 665 correctly predicted loops in the CTCF dataset, achieving an accuracy of 22%. Mustache predicted 939 loops, with 543 correctly predicted loops in the CTCF dataset, attaining the highest accuracy of 58%. CD-Loop had the highest number of successfully predicted loops and ranked second in accuracy, while Mustache, with the highest accuracy, had the fewest successfully predicted loops, only half of CD-Loop’s count. Specific data for RAD21 can be found in Figure 3C. CD-Loop has advantages over other tools, being able to identify more loops supported by CTCF and RAD21, demonstrating good performance in both precision and recall.

Stacking analysis of surrounding CTCF-binding sites at predicted chromatin loop anchor locations is shown in Figure 3D, indicating that the chromatin loops predicted by these four methods are rich in CTCF-binding motifs and have little difference, indicating that the same training model can not only identify CTCF motifs in GM12878 cell lines but also be applicable in K562 cell lines. Similar results were obtained on IMR90 data (Figure 3E). Whether it is the K562 cell line or IMR90 cell line, the number of CTCF-binding sites at the left and right anchor points of the chromatin loop detected by CD-Loop is less than that of the other three methods. However, the number of CTCF-binding sites present was higher than other methods, within a 50-Kb range of the left and right anchor points of the chromatin loop. The reason may be that we represent a chromatin loop connected by multiple pixels as a single pixel as a single chromatin loop. Due to the expansion of the range of the anchor point of the chromatin loop, the CTCF-binding sites present on the anchor point are also within a certain range float.

#### 3.2.3 Specificity analysis

In addition, to further illustrate the differences between cell lines, we conducted a comparison of chromatin loop overlap among three cell lines; to enhance fault tolerance, we allowed partial matches (±5 kb) between any anchors in two bins. As shown in Figure 3F, even when the overlap range was increased, the extent of the chromatin loop overlap was relatively low among the three cell lines, suggesting that the chromatin loops are specific to each cell type.

#### 3.2.4 mESC cell line enrichment experiments and CTCF-binding site analysis

Enrichment experiments and CTCF ChIP-Seq signal analysis for mESC data are shown in Figure 3G, H. In the CTCF ChIA-PET enrichment experiment, CD-Loop predicted the most loops among the four methods, and the number of overlaps increased linearly with the increasing number of predicted loops. The number of perfectly matched CTCF-binding sites is slightly lower compared with the other three methods, but the number of CTCF-binding sites around the anchor fluctuation range was higher than the other three methods.

In conclusion, the research results show that CD-Loop has achieved superior performance in human K562 and IMR90 cell lines and mouse cell types.

### 3.3 Experimental results at different sequencing depths

#### 3.3.1 Quantity, F1-score, and enrichment experimental analysis

**FIGURE 4:**
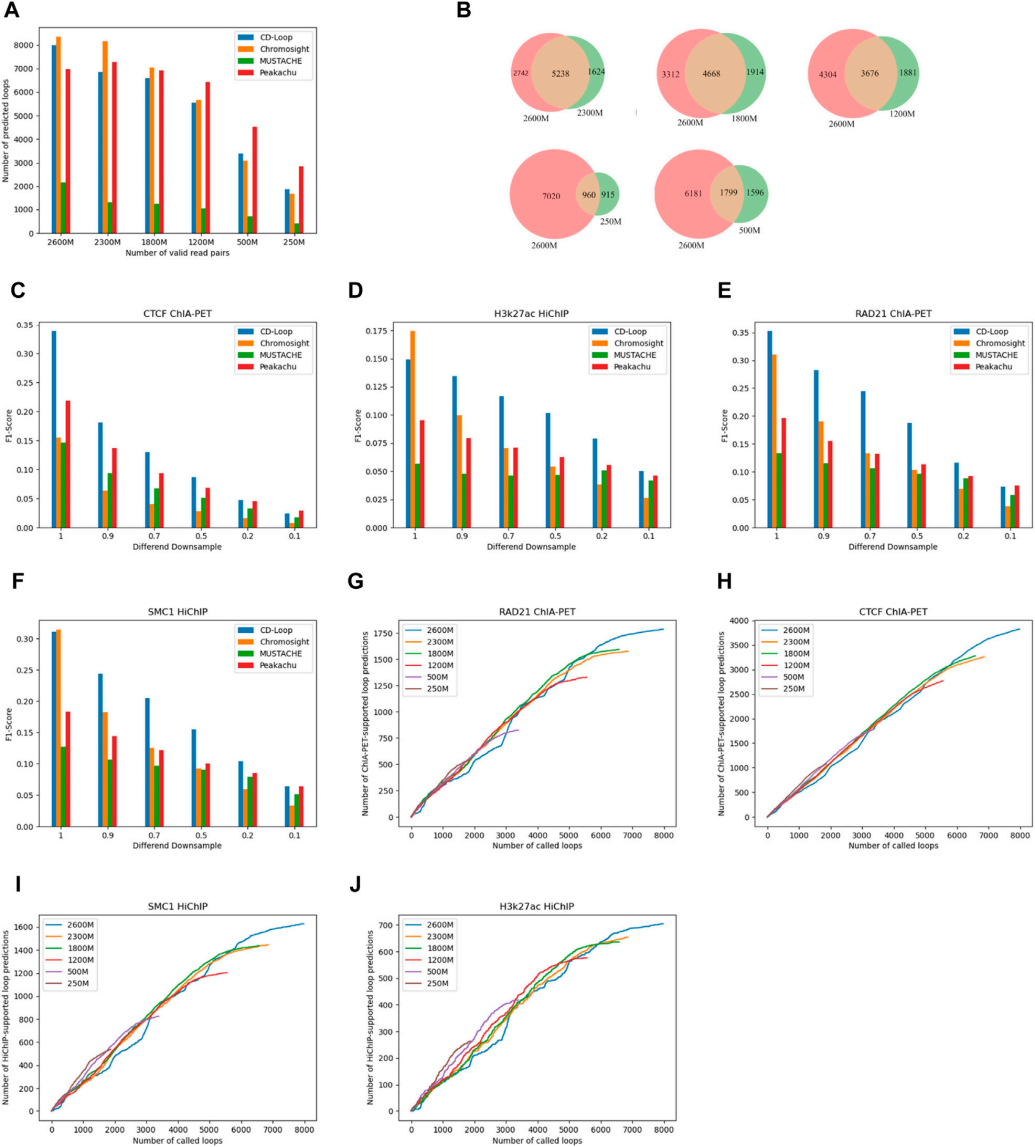
Evaluation at different sequencing depths. **(A)** The number of chromatin loops predicted by different methods decreases as the number of effective chromosome read pairs decreases. **(B)** Venn diagrams at different sequencing depths. **(C–F)** F1-scores of different enrichment experiments, including CTCF ChIA-PET **(C)**, H3k27ac HiChIP **(D)**, RAD21 ChIA-PET **(E)**, and SMC1 HiChIP **(F),** in GM12878 cells at different sequencing depths. **(G–J)** Number of supports on different enrichment data [RAD21 ChIA-PET **(G)**, CTCF ChIA-PET **(H)**, SMC1 HiChIP **(I)**, and H3k27ac HiChIP **(J)**] for predicted chromatin loops at different sequencing depths.

To evaluate the ability of CD-Loop at different sequencing depths, we conducted downsampling experiments using the FAN-C method (Kruse et al., 2020)on the original sequencing depth of 2,600 M valid read pairs. We performed downsampling at various percentages, including 90%, 70%, 50%, 20%, and 10%. The corresponding effective read pairs for each downsampling were 2,300 M, 1,800 M, 1,200 M, 500 M, and 250 M. Using default parameters for different loop prediction tools, we observed a decrease in predicted chromatin loops as the sequencing depth decreased, as shown in Figure 4A. CD-Loop and Peakachu predicted the highest number of chromatin loops. However, in enrichment experiments (Figures 4C–F), CD-Loop consistently achieved the highest F1-score among the four methods. The F1-score decreased with decreasing sequencing depth but remained at its highest level. The enrichment experiments for different methods at different sequencing depths are shown in Supplementary Figure S1 in Supplementary Material.

#### 3.3.2 Robustness analysis

We assessed the overlap between the loops predicted by CD-Loop at different sequencing depths and the loops present in the original sequencing depth matrix. As depicted in Figure 4B, the overlap rates were 76%, 68%, 66%, 54%, and 51% for downsampling matrices with 2,300 M, 1,800 M, 1,200 M, 500 M, and 250 M valid read pairs, respectively. This high overlap rate indicates that CD-Loop not only predicts a significant number of chromatin loops but also detects more loops at low sequencing depth. Moreover, it will not cause more false positives, highlighting the robustness of CD-Loop.

#### 3.3.3 Sensitivity analysis

CD-Loop efficiently identifies a significant quantity of loop structures within sparse data without increasing the number of false positives. As shown in Figures 4G–J, when evaluating loops mediated by CTCF, RAD21, SMC1, and H3K27ac in low-depth datasets, CD-Loop maintains a high level of accuracy. This implies that the predictions made for low-sequencing depth data are almost as accurate as predictions on complete data, with lower sensitivity.

Overall, CD-Loop outperforms other tools in terms of accuracy at all sequencing depths. These results highlight the superior robustness, accuracy, and reliability of CD-Loop.

### 3.4 Hyperparameters and resolution analysis

In order to prove the generalization ability of CD-Loop, we conducted different experiments on the three hyperparameters of the optimizer, batch size and epoch, and used chromosome 15 as the test set to verify the optimal hyperparameters of the model. The experimental results are shown in Supplementary Tables S1–S3 in Supplementary Material.

In addition, we used chr15 as the test set and conducted experiments at three resolutions: 5 KB, 10 KB, and 25 KB. The experimental results are shown in Supplementary Table S4 in Supplementary Material.

## 4 Discussion

Here, we propose CD-Loop, a deep learning-based method that uses diffusion models to predict the chromatin loops from a given Hi-C contact map. Our extensive evaluations indicate that CD-Loop outperforms existing tools in loop annotation for datasets with various sequencing coverages.

The main contributions of CD-Loop are as follows: 1) the development of a deep learning framework that first conducts pre-training to obtain prior probabilities and then utilizes the denoising process of the diffusion model and a pre-trained estimative model for forecasting chromosomal loops in Hi-C contact matrices, resulting in improved accuracy for genome-wide chromatin loop recognition; 2) the use of data augmentation by flipping the interested parts of the Hi-C matrices in all four directions, which increases the diversity of training data and improves the generalization ability of the model, allowing for the training of a unified framework designed for processing Hi-C datasets from different sequencing depths, cell types, and species. A series of experimental results demonstrate that CD-Loop can effectively improve chromatin loop recognition accuracy compared to other methods and identify a range of unique chromatin loops. The overlap rate between different sequencing depths within the same cell line is relatively high, while the overlap rate between different cell lines is relatively low. Finally, equally important is that CD-Loop exhibits good robustness and stability on different biological cells and sequencing depths.

Although CD-Loop has superior performance compared to other methods, there are still areas that need optimization and improvement: 1) when predicting the entire Hi-C matrix, the prediction time is long. It can be improved by processing the data to reduce the waiting time. 2) CD-Loop can also be extended to analyze data at a higher resolution, but this would require optimizing the data processing procedure to reduce memory usage and IO time.

CD-Loop is a method that implements three-dimensional genome data analysis based on diffusion model classification. It enables accurate prediction of Hi-C contact maps at medium sequencing depth and improves the accuracy of its analysis even at low sequencing depth. With the continuous increase in high-quality Hi-C datasets, we expect that the capabilities of CD-Loop will be further improved and developed.

## Data Availability

The original contributions presented in the study are included in the article/Supplementary Material; further inquiries can be directed to the corresponding author/s.
