# Supplementary material for: CD-Loop: a chromatin loop detection method based on the diffusion model
Source: Front Genet. 2024 May 6;15:1393406. doi: 10.3389/fgene.2024.1393406 (PMC11102972; doi:10.3389/fgene.2024.1393406)
Supplement: Supplementary file 1 [file Table1.DOCX]

**Supplementary Materials**

CD-Loop: A chromatin loop detection method based on the diffusion model

1. Comparison under different sequencing depths


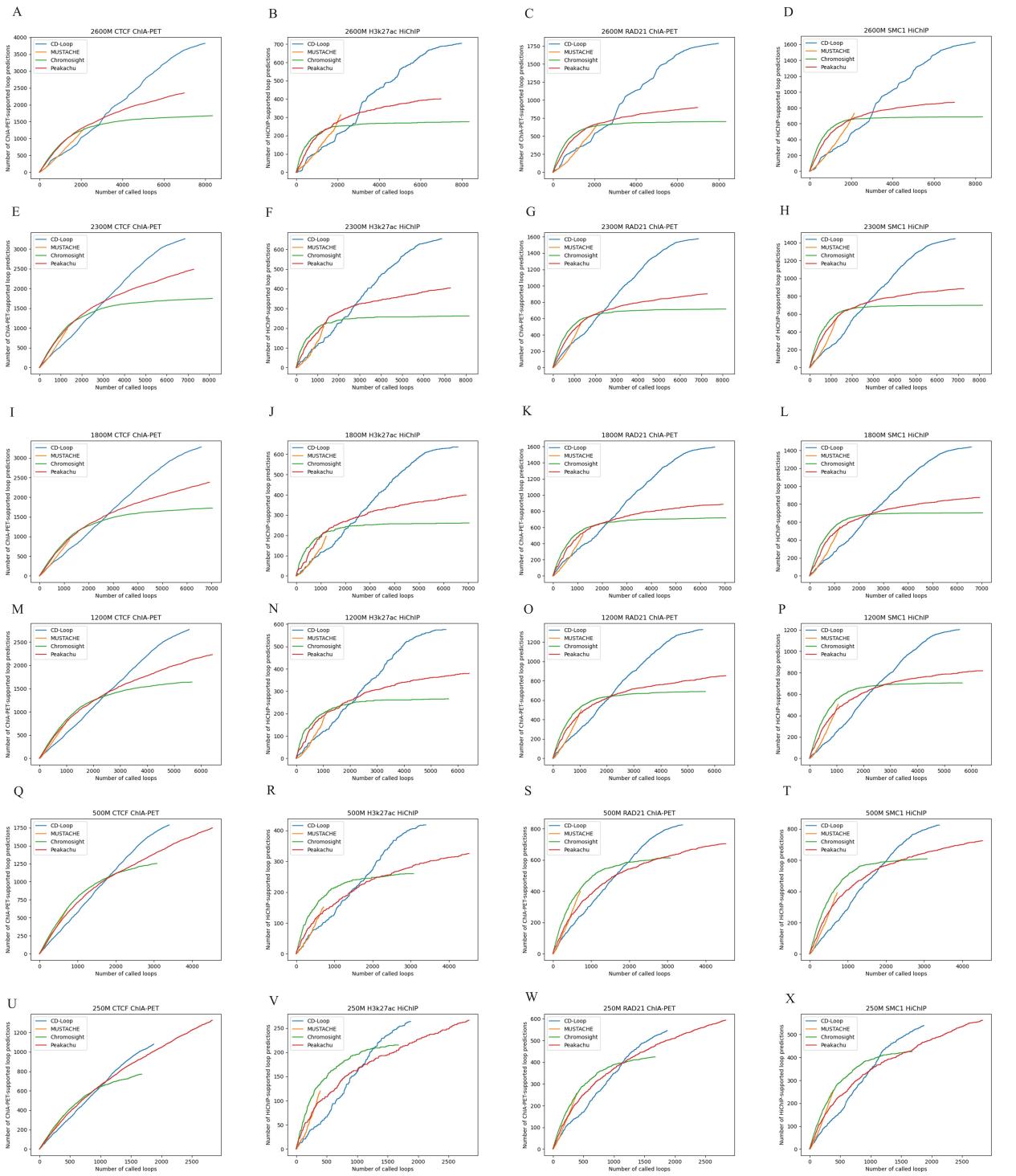


Figure S1. Comparison of CD-Loop, Chromosignt, Peakachu, and Mustache on GM12878 Hi-C data with different sequencing depths (2600M, 2300M, 1800M, 1200M, 500M, 250M). The comparison is performed on four enrichment experiments including CTCF ChIA-PET, RAD21 ChIA-PET, H3K27ac HiCHIP, and SMC1 HiCHIP on chromosomes 15-17.

2. Hyperparameter analysis

In order to prove the generalization ability of CD-Loop, we conducted different experiments on the three hyperparameters of optimizer, batch size and epoch, and used chromosome 15 as the test set to verify the optimal hyperparameters of the model. The experimental results of different optimizer are shown in Table S1, the experimental results of different batch size are shown in Table S2, and the experimental results of different epoch are shown in Table S3.

Table S1. Experimental results of different optimizer

|  | recall | precision | accuracy | F1-score |
| --- | --- | --- | --- | --- |
| Adam | 0.8665 | 0.8747 | 0.9352 | 0.8706 |
| RMSProp | 0.8282 | 0.9007 | 0.9338 | 0.8629 |
| SGD | 0.8592 | 0.8814 | 0.9355 | 0.8702 |
| NAdam | 0.8747 | 0.8923 | 0.933 | 0.8834 |

Table S2. Experimental results of different batch size

|  | recall | precision | accuracy | F1-score |
| --- | --- | --- | --- | --- |
| 64 | 0.8612 | 0.8701 | 0.9327 | 0.8656 |
| 128 | 0.85 | 0.8782 | 0.9326 | 0.8639 |
| 256 | 0.8747 | 0.8923 | 0.933 | 0.8834 |

Table S3. Experimental results of different epoch

|  | recall | precision | accuracy | F1-score |
| --- | --- | --- | --- | --- |
| 100 | 0.8747 | 0.8923 | 0.933 | 0.8834 |
| 90 | 0.8508 | 0.8783 | 0.9329 | 0.8643 |
| 80 | 0.8572 | 0.8747 | 0.9333 | 0.8659 |
| 60 | 0.856 | 0.8827 | 0.9353 | 0.8692 |
| 40 | 0.8560 | 0.8752 | 0.9331 | 0.8655 |

In terms of optimizer parameters, we selected four optimizers: Adam, RMSProp, SGD, and NAdam for comparison. As shown in Table S1, we can conclude that the effect of the NAdam optimizer is better than the other three methods, so we chose NAdam as the ultimate optimizer. Regarding the selection of batch size, we conducted experiments at 64, 128, and 256 respectively. From Table S2, it can be concluded that 256 is the optimal result, so the final batch size is 256. Regarding the size of the epoch, we conducted the 100th, 90th, 80th, 60th, and 40th rounds of testing respectively. From Table S3, it can be concluded that when the epoch is 100 rounds, the test results have the optimal performance, so we finally set the epoch size to 100.

3. Resolution analysis

We used chr15 as the test set and conducted experiments at three resolutions: 5KB, 10KB, and 25KB. The experimental results are shown in Table S4. As the resolution decreases, different evaluation indicators also decrease. From the table, we can see that as the resolution decreases, different evaluation indicators decrease, and the accuracy remains at around 90%. From this, it can be concluded that the resolution of the Hi-C contact map will affect the final detected chromatin loops.

Table S4. Experimental results at different resolutions

|  | recall | precision | accuracy | F1-score |
| --- | --- | --- | --- | --- |
| 5KB | 0.8747 | 0.8923 | 0.933 | 0.8834 |
| 10KB | 0.8036 | 0.8406 | 0.9101 | 0.8217 |
| 25KB | 0.7782 | 0.7816 | 0.891 | 0.7789 |
